# Role of laissez-faire leadership in talent management: Evidence from the pharmaceutical industry of Bangladesh

**DOI:** 10.1016/j.heliyon.2023.e17234

**Published:** 2023-06-15

**Authors:** Mohammad Ali, Muhammad Shariat Ullah

**Affiliations:** a Department of Business Administration in Management Studies, Bangladesh University of Professionals (BUP), Dhaka, Bangladesh; b Department of Organization Strategy & Leadership, Faculty of Business Studies, University of Dhaka, Bangladesh

**Keywords:** Laissez-faire leadership, Talent management, Talent attraction, Talent retention, Talent engagement, Talent development

## Abstract

Laissez-faire leadership is mainly perceived as zero leadership, and research on it is relatively scant compared with other dominant approaches to leadership. Although the adverse effects of laissez-faire leadership have been well examined, its influence on talent management (TM) has been undiscovered. This study assessed the impact of laissez-faire leadership on TM strategies, including talent attraction, retention, engagement, and development. Data were collected from 460 employees of pharmaceutical companies in Bangladesh using judgmental sampling. Structural equation modeling was employed to test the hypothesized relationships between laissez-faire leadership and TM strategies using social exchange theory. This study found positive effects of laissez-faire leadership on talent attraction, retention, development, and engagement. These findings suggest that if talented employees are given freedom, they tend to engage and secure more opportunities for self-directed development by solving problems independently. This study contributes to the understanding of how laissez-faire leadership may constructively affect TM in the context of a developing economy's pharmaceutical industry. Finally, this study provides recommendations for practitioners of pharmaceutical companies to improve their strategic choices regarding laissez-faire leadership to ensure better TM strategy practices.

## 1 Introduction

The demand for leadership responsibility in managing talent has gained scholarly attention in business studies [43]. Many studies have endeavored to impart insights on the critical antecedents of talent management (TM), such as perceived organizational justice [41], organizational culture [57], managerial support [59], leadership [15,53], transformational leadership [97,104], inclusive leadership [50], thereby enlightening our comprehensiveness about the mechanism of improving TM strategies in the organization. However, perceiving the negative aspects, less attention has been paid to laissez-faire leadership; hence, more studies should investigate the impact of laissez-faire leadership on TM. Owing to its potential constructive outcomes for managing educated young talents, it warrants in-depth empirical exploration [101].

Laissez-faire leadership is included in the full-range leadership model [8], one of the most established and popular models [88]. Although organizational leaders frequently use laissez-faire leadership, this approach has received little attention from researchers [31], leading to a dearth of empirical evidence on laissez-faire leadership because constructive leadership is prioritized to ensure better management of talents in organizations [103], such as transformational [97], inclusive [50], ethical [81], and servant leadership [36]. Only few studies have assessed laissez-faire leadership and its effect on followers’ behavioral outcomes, such as employee commitment [1], affective commitment [88], employee engagement [101], employee motivation [111], and employee performance [32]. However, the findings of previous studies have been mixed and inconclusive.

Some studies argue that laissez-faire leadership can be characterized as absent leadership because the leaders extend little or no intervention in decision-making [51,88]. Laissez-faire leadership differs from transactional and transformational leadership, and it is presumed ineffective [108]. Moreover, most previous studies [24,31,32,39,70,77,84] found negative effects of laissez-faire leadership on employee behavior, leading to a one-dimensional negative view and debate on the outcomes of practicing laissez-faire leadership.

Laissez-faire leadership may not always show ignorance, carelessness, or indifference toward the needs of the subordinates [96] as mostly perceived. For instance, leaders may engage in many activities besides monitoring subordinates' performance and subordinates may positively accept being left alone to manage their activities [108]. As a hands-off approach, laissez-faire leadership creates a culture of autonomy and self-belief that might have positive effects owing to leaders' low or non-involvement [108]. Moreover, leaders' excessive involvement limits followers' innovation propensity [90]. Laissez-faire leaders pave the way for employees to make decisions and work at their convenience [101], thus instigating employees' self-development to solve difficulties in the tasks. Laissez-faire leadership enhances employee's innovation and creativity by providing autonomy to execute decisions without delays or waiting for approval [5]. However, the suitability of a laissez-faire leadership approach depends largely on the extent to which employees are self-disciplined, responsible, skilled, and learned. Apart from the contentious findings on the relationship between laissez-faire leadership and employees' behavioral outcomes, previous studies did not empirically focus on justifying the relationship between laissez-faire leadership and TM. Therefore, the present study focuses on laissez-faire leadership to address this gap and examines its impact on TM in emerging economies, such as Bangladesh.

TM has gained attention because it promises to facilitate talent capacity building [60] and supports organizational viability [72]. TM practices including employee devotion, engagement, and development are required to gain sustainable competitive advantages [30,74]. Eva [38] argued that to gain competitive advantages, organizations need to develop short and long-term plans to attract, retain, and develop talented people and position them in the right place at the right time for the right jobs. TM has been gaining importance as managers and leaders are interested in investing more time and resources in acquiring and developing a talented workforce [3,60]. Talent is crucial for achieving organizational objectives and meeting business demand [46,73]. Talented employees can accomplish extraordinary outcomes, and they are regarded as an organization's top performers [67,99]. Nijs et al. [79] stated that the rapid pace of innovative improvement, globalization, and crossing the boundaries of nations prompt the present circumstance wherein everyone comprehends the genuine estimation of talent. Hence, it is important to apply and practice TM strategies to ensure the attraction, retention, development, and maximum utilization of people in organizations.

This study applies social exchange theory (SET) as a theoretical lens to illustrate the relationship between laissez-faire leadership and TM strategies. The SET assumes that reciprocity between leaders and followers can influence followers' behavioral outcomes in the workplace. A leader's approach can affect TM practices as an essential part of organizational strategy [11]. Laissez-faire leaders empower employees and increase their self-determination, self-competence, and autonomy. Employees feel obligated to return by demonstrating an intention to stay longer, encouraging others to stay or choose the organization, a higher level of work engagement, and dedication to self-development. Previous studies have used SET to integrate different relationships between leadership and TM, such as transformational leadership, employee engagement, organizational change initiatives [47], TM, and retention intention [61]. However, few studies have attempted to apply the SET to integrate the framework of laissez-faire leadership with TM strategies. Therefore, this study assesses the relationship between laissez-faire leadership and each of the four components of TM: talent attraction, retention, engagement, and development.

The present study focuses on the pharmaceutical industry of Bangladesh, as it is one of the most prominent industries in the country and has seen tremendous growth over the years in local and international markets. More than 125 countries import pharmaceutical products from Bangladesh, and this industry meets nearly 98% of the domestic demand [80]. Therefore, this growing sector requires a talented workforce and appropriate strategies to manage talent and facilitate sustainable competitive advantages. The contributions of this study to TM and leadership are two-fold. First, it addresses the role of laissez-faire leadership in TM. Earlier studies in the pharmaceutical industry focused on various leadership styles, such as authentic [98], ethical [35], servant [21], transformational [76,78], autocratic [7], technological [6], strategic [4], green [49], green transformational [62], and market leadership [69]. Limited focus has been placed on the impact of laissez-faire leadership on the TM outcomes of pharmaceutical organizations [108]. In the present context, pharmaceutical leaders' laissez-faire approach and its linkage with TM strategies have yet to be highlighted in the earlier research [38]. Second, this study can be a building block for reconsidering the development and application of laissez-faire leadership. The findings of the study suggest that young talents in the present context want freedom, autonomy, and less intervention in their tasks [38]. Thus, this study provides new insights into how employees perceive a laissez-faire approach and connects it with the strategies of TM.

**Fig. 1:**
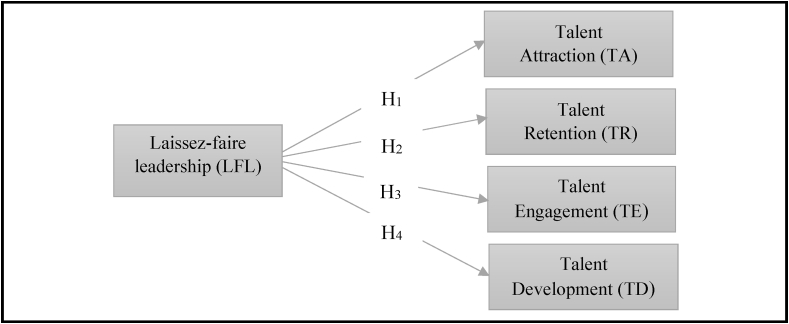
Research framework.

The paper is structured as follows. It starts with the discussion on the importance of TM and the role of leadership in implementing TM. We demonstrate literature on social exchange theory, TM strategies and laissez-faire leadership. Next, we present the development of the hypotheses (Fig. 1), followed by the methodology, results, and discussion. We then discuss the theoretical and practical implications. Finally, limitations and future research directions are offered, along with conclusions.

## 2 Literature review

### 2.1 Social exchange theory

SET integrates and justifies the research framework for this study. Blau [16] conceptualized social exchange as the voluntary actions of individuals motivated by the mutual returns they expect to receive and deliver to others. SET is a popular management theory that describes leadership approaches and employees’ behavioral outcomes in an organization [100]. This theory assumes that resource exchange occurs during the interaction between the two parties [17]. Since social exchange is a two-way transaction that necessitates both giving and receiving, it is regarded as involving interdependence between participants [27]. Even though the nature of reciprocation is unclear, social exchange occurs when interactions between two parties give rise to a sense of duty to reciprocate one another [16]. The reciprocation behaviors that are prompted to return favors to the initiating party determine the “rules” that must be followed in an exchange process to establish an exchange relationship [27].

This study argues that laissez-faire leaders, who provide freedom and autonomy to employees and ensure an independent work environment, initiate the social exchange process. When employees recognize the favors demonstrated by leaders, they reciprocate with higher engagement, more extended attachment, more dedication to self-development, and sharing positivity about the organization in order to return the favor. Employees who enjoy the benefits of flexibility and delegation from laissez-faire leadership behaviors are encouraged to learn, be attentive, and engage in their tasks. The interpersonal connections between the two exchange parties are supported by the continuation of exchange favors with the same party [26]. Consequently, laissez-faire leaders continue to provide freedom, autonomy, delegation, and the authority to receive reciprocation from employees.

### 2.2 Talent management strategy

TM refers to strategies that attract, retain, engage, and develop individuals with skills and knowledge valuable to an organization [82,109]. It is a deliberate approach to attract, retain, engage, and develop people with the skills required to meet an organization's current and potential needs [71]. The first strategy used in this study is talent attraction. Talent attraction is a systematic way of presenting an organization to people so that jobs look appealing and qualified people are encouraged to apply and be proud to be part of the organization. Attracting employees with the required skills is a prime concern for organizational leaders [12,29,107]. Employer branding is significant in attracting the right talent and includes the image and reputation of the organization [18,87]. In addition, talent retention, a strategy for retaining valued employees for longer periods [87,95], primarily aims to reduce employee turnover and improve productivity and service quality [68]. Talent retention is essential for companies to survive in a competitive labor market [95,107].

Talent engagement is another essential TM component. This involves physical and psychological involvement in the job [54]. The physical efforts of talented employees and their feelings toward their job and organization are essential for better performance. Witemeyer [105] stated that engaged employees are involved in sharing knowledge, proactive problem solving, collaboration with peers, and decision-making processes. Engaged employees are more efficient and productive [91,94], they think beyond their primary responsibility, and try to exert maximum effort to achieve organizational goals [23]. An organization's performance improves if employees become engaged and devoted to innovation and creativity, dedicated to responsibility, and prioritized emotionally [94]. Another element of TM is talent development, which focuses on developing an existing talent pool to reap the maximum business return over an extended period [28,86]. Talent development is significant, as talent intends to improve company-specific relevant skills [25,28]. Thus, talent development is a dominant strategy of TM, an ongoing approach to improve the caliber of existing talent to execute current assignments and cope with potential changes for the betterment of the organization. Recognizing the importance of TM, organizational leaders deploy various TM strategies to sustain a volatile competitive business environment. Hence, the leadership approaches adopted by organizational leaders are crucial for implementing TM strategies.

### 2.3 Laissez-faire leadership

The extent of effective implementation of TM strategies depends on leaders' behavioral aspects at different levels of an organization. Leaders must focus on the engagement and development of talent and evoke the potential of people to succeed in competitive business environment [101]. Mathew [71] found that leadership and succession planning are highly integrated with managing talent across organizations. Leadership refers to an individual's ability to influence others to be enthusiastic about accomplishing objectives [20]. Leadership is a process that influences and stimulates subordinates to achieve organizational goals. Leaders apply various leadership styles, even in the same organizational environment. Laissez-faire leadership is a leadership approach that has received less attention to research than other leadership styles [88].

When effective or no evidence of leadership is absent, it can be referred to as laissez-faire leadership [110]. Laissez-faire leaders avoid problems, are less aware of decision-making, dislike feedbacks, and refuse to be involved [31,101]. Leaders rarely interact with group members, prefer to avoid taking responsibility, or play passive roles in team activities [32,66]. Further, the leaders do not impart clear instructions to their followers. In most cases, leaders ignore challenges and problems [110] and do not emphasize rewards or feedbacks regarding employees’ performance. Laissez-faire leaders delegate most decisions to their subordinates [111]. In addition, subordinates lack guidance and supervision, and receive little support from laissez-faire leaders [32,66]. Consequently, laissez-faire leadership is positively associated with job stressors such as conflicts with colleagues, ambiguity of roles, and vague responsibilities [96].

However, Yang [108] stated that laissez-faire leadership is not zero leadership and should be treated beyond conventional context-based presumptions; such a leadership approach can bring positive outcomes because leaders who adopt this style provide autonomy and freedom to their subordinates. Subordinates can make decisions using the necessary resources and materials [101]. It is expected that followers will solve their problems; subsequently, they will secure learning opportunities to develop themselves [31,33]. Laissez-faire leadership is considered a practical approach only when the followers are highly skilled, knowledgeable, capable, and intend to do it independently [24,101]. This leadership style can also be effective when subordinates’ tasks are programmed, recurring, and routine, and when the nature of the decision is not complicated but instead predetermined [111].

Hence, laissez-faire leadership is inappropriate when subordinates lack skills and expertise, and the job is non-recurring and complex. In addition, when subordinates are reluctant to make decisions and to willingly get involved in performing assigned tasks, this leadership style may not be compatible. If employees do not have the caliber to make decisions and initiatives, laissez-faire leadership may fail and cannot bring success to the organization. Therefore, contextual and moderating factors should be considered to predict the positive or negative outcomes of laissez-faire leadership.

## 3 Hypotheses

### 3.1 Laissez-faire leadership and talent attraction

Attracting talent is associated with some factors [18,29] influenced by laissez-faire leadership. Attracting talent largely depends on an organization's reputation [29], whereas reputation is associated with employee satisfaction. However, Bass and Avolio [14] stated that leaders who follow the laissez-faire approach rarely attempt to identify and satisfy subordinates' needs or motivate them. Bass [13] reported no direct relationship between laissez-faire leadership and employee attraction, whereas Judge and Piccolo [52] and Robert and Vandenberghe [88] found a negative association between laissez-faire leadership and job satisfaction and attracting qualified employees. Such leadership creates stress for employees [55,88], which may also be one reason for its weakness in attracting talent. Laissez-faire leadership, as a non-strategic leadership approach, does not contribute significantly to attracting talent [45].

However, Yang [108] found the opposite outcome: autonomy may enhance employee motivation and increase the probability of attracting talent. Moreover, the opportunity to think independently and creatively, which is a determinant of talent attraction, is positively related to the passive approach to leadership [90]. Laissez-faire leaders enhance the extent of talent attraction as they are aligned with SET, where the independence and autonomy provided by leaders lead to the reciprocation in the form of dispersion of positivity within and outside the organization, which attracts others. Laissez-faire leaders empower employees [106] and create an attractive climate. Therefore, we postulate the following hypothesis:

Hypothesis 1 Laissez-faire leadership is positively related to talent attraction.

### 3.2 Laissez-faire leadership and talent retention

As laissez-faire leaders are not highly interested in making decisions or involving themselves in decision-making, they hardly contribute to satisfying subordinates, leading to turnover intention [110]. Consequently, talented employees do not receive sufficient support or career guidelines to fulfill their career growth aspirations. Consequently, talent tends to switch to a better career and sees more explicit career paths. Poor communication and indifference to involvement in decision-making lead to employee dissatisfaction and decrease employee retention [39]. Hence, it is difficult for laissez-faire leaders to retain talented employees for longer periods. Ahmad et al. [2] found that laissez-faire leadership positively relates to employee turnover intention.

However, it is also a matter of concern that talented employees want job freedom and the autonomy [75] to make decisions and perform tasks; such determinants are present in laissez-faire leadership. Yang [108] points out that an autonomy-based leadership approach cultivates more determination toward work, enhancing the possibility of retaining talent in an organization. Retention possibility also increases because of the innovation opportunities, which depend mainly on the extent of leaders' involvement in employees’ affairs [90,95]. Laissez-faire leaders provide job flexibility and decision-making authority, and employees reciprocate these benefits by serving the organization longer with higher loyalty. Therefore, this study hypothesizes that:

Hypothesis 2 Laissez-faire leadership is positively related to talent retention.

### 3.3 Laissez-faire leadership and talent engagement

Under the laissez-faire leadership approach, leaders are not highly engaged in tasks; therefore, expecting subordinates to be highly engaged in their work may be challenging. Since laissez-faire is a less active leadership approach, subordinates enjoy greater freedom and control over their jobs. Nevertheless, followers have more control under this approach and are not highly motivated to devote additional efforts [102]. Previous studies [70,77,84] have reported a negative association between laissez-faire leadership and subordinates’ behaviors, as laissez-faire leaders avoid problems. Nelson and Shraim [77] found that when leaders adopt laissez-faire leadership behavior, their degree of work engagement in the organization declines. Popli and Rizvi [84] supported the negative relationship between the laissez-faire style and engagement, as leaders show less interference and avoid decision-making. Passive avoidant leadership is negatively related to service orientation and employee engagement [85]. Manning [70] also supported the negative impact of laissez-faire leadership on nursing staff engagement owing to a lack of feedback and delays in decision-making.

However, Yang [108] stated that leaders' non-involvement fosters high self-control, determination, and followers' engagement with their work. Followers' autonomy positively influences the concentration and persistence in work [65]. Laissez-faire leadership enhances employees' propensity to innovate and self-engage, as less of leader's involvement facilitates thinking independently, with a positive psychological attachment to the job [90]. Laissez-faire leaders allow employees to think and make decisions creatively without intervention; thus, they can take critical responsibilities [101]. Thanh and Quang [101] found that laissez-faire leadership significantly affected employees' work engagement. Talent engagement is argued to be the reciprocating behavior of talent toward laissez-faire leaders [101]. Therefore, this study hypothesizes the following:

Hypothesis 3 Laissez-faire leadership is positively related to talent engagement.

### 3.4 Laissez-faire leadership and talent development

Empirical evidence on laissez-faire leadership demonstrates a negative association with employees' attitudes and toward employees' development [52]. Skogstad et al. [96] postulate that laissez-faire leadership is a destructive leadership approach because it generates ambiguity, role conflict, and worker disagreement. Laissez-faire leadership is indirect and passive; hence, it does not focus on subordinates' development [96]. The lack of clear guidelines for performing a job leads to employee's frustration, which negatively influences the skills [34]. Laissez-faire leaders do not significantly motivate followers to develop skills to effectively perform a job [24]. Typically, laissez-faire leaders are barely involved in work units and making decisions; hence, there is a lower likelihood for followers to learn something directly from leaders [24]. Khan et al. [56] found that laissez-faire leadership negatively influences innovative work attitudes. They concluded that laissez-faire leaders, who are inactive, tend to delay decision-making. Laissez-faire leaders avoid responsibilities and focus less on the development of subordinates.

Yang [108] presented an alternative finding to demonstrate the positive outcomes of laissez-faire leadership. When leaders leave their subordinates alone, employees experience autonomy and freedom in their work, which foster self-development by solving problems without leaders' interventions [108]. Ryan and Tipu [90] found that laissez-faire leadership enhances subordinates’ innovative attitudes and facilitates a creative working environment. Eagly et al. [33] claimed that when subordinates enjoy freedom and solve problems, opportunities to learn and develop are enhanced. Thanh and Quang [101] argued that laissez-faire leaders allow followers to face difficult situations and solve critical problems. Thus, employees learn to enhance their personal growth by performing complex tasks. When talents observe that laissez-faire leaders provide independent responsibility and appreciate through rewards and recognition, they reciprocate by demonstrating a greater inclination to learn and develop for better performance. Hence, we formulate the following hypothesis:

Hypothesis 4 Laissez-faire leadership is positively related to talent development.

## 4 Method

The study was cross-sectional as data were collected at one point [93] and conclusions were drawn through examination at a specific time. This study used a quantitative approach in which a structured questionnaire was employed to collect data from respondents. The questionnaire method is logical for studying sociological constructs, such as leadership and managerial practices [92]. The data used in this study include laissez-faire leadership and the extent to which TM strategies are being practiced.

### 4.1 Sample

It is essential to know the population of any study, as it is the entire group of people the researcher wants to investigate [89,93]. Misguidance is possible if the population is not correctly defined. However, no clear or specific information is available regarding the total number of employees in the pharmaceutical industry. The labor force survey (2016–2017) of the Bangladesh Bureau of Statistics presented an approximate total number of employees. Therefore, the approximate population for this survey was 1,77,000 [10,48]. The population for the current study comprises of employees working in pharmaceutical companies in Bangladesh.

The sample consists of 460 employees from Bangladesh's pharmaceutical industry. Bangladesh is an emerging economy with a GDP growth rate of more than six percent over the last ten years, where leaders' perceptions of a less focused leadership approach need to be addressed. This study focused on the pharmaceutical industry for two reasons. First, the rapid growth and expansion of this sector at 15% year-on-year growth rate to reach $5.11 billion by 2023, making it the country's second largest industry in earning foreign currency and contributing to the national exchequer [48]. Second, this industry is very competitive, and managing talent is becoming a matter of competition for organizations, as they compete for the same talent pool. Therefore, this study examines the emerging economy's pharmaceutical industry to measure the role of laissez-faire leadership in TM.

This study employed judgmental sampling, a nonprobability sampling technique, to collect data from employees of the pharmaceutical industry in Bangladesh. Judgmental sampling has been suggested as an expert and educated guess to represent any target population [19,43]. Judgmental sampling occurs when a researcher uses a sample that conforms to some criteria [112]. In the current study, 32 companies were selected based on two criteria: (a) the firm employed at least 100 employees and (b) it was listed on the Dhaka Stock Exchange.

A written request for data collection was sent to the human resources departments of the selected pharmaceutical companies. Informed consent was obtained from all the participants upon approval of the survey. A self-administered questionnaire was delivered to 700 employees who fulfilled two criteria: (a) had at least one year of job experience in the company, and (b) worked under a direct supervisor. With the support of the Human Resources Department, 460 completed questionnaires were returned and the response rate was approximately 66%. Almost two months were required to gather data, from November to December 2021.

### 4.2 Measures

Twenty-nine items were used to design the research instrument, all of which were adapted. Three items developed by Bass and Avolio [14] were used for measuring laissez-faire leadership (e.g., *“My immediate boss avoids making decisions”*) through a 5-point Likert scale ranging from 1 (Not at all) to 5 (Frequently). Seven items developed by Highhouse et al. [44] measured the extent of talent attraction, and five items were adapted from Kyndt et al. [63] to measure talent retention. Talent development was measured using five items adapted from the work of Chami-Malaeb and Garavan [22]. Talent engagement was measured using nine items developed by Bakker and Schaufeli [9]. Sample items of the TM strategies included “*The jobs of my company are appealing to people”* (talent attraction); *“I have the plan to work for this company at least for five years or as long as I can”* (talent retention); *“My company arranges seminars, workshops, and conferences for skill development of employees”* (talent development); *“At my work, I feel bursting with energy”* (talent engagement). All TM strategy items were measured using a 5-point Likert scale ranging from 1 (strongly disagree) to 5 (strongly agree).

Since the data were collected from a single source, there may be a risk of common method variance (CMV). Hence, the Harman single-factor test was used to assess the likelihood of common method variance [83]. The first factor accounted for only 39.63% (<50%) of the total variance. This finding implies that the proposed model is not affected by the CMV.

### 4.3 Analytical approach

Descriptive statistics were calculated using Statistical Package for Social Sciences. The research model was assessed in three stages. The first stage included model fitness. In the second stage, the reliability and validity were measured. In the third stage, the hypothesized relationships were tested with Structural Equation Modeling (SEM) using Analysis of Moment Structures (AMOS) software. SEM is a second-generation data analysis technique that assesses the linkages between multiple constructs [42]. The measurement and structural models are two inseparable parts of the AMOS SEM. The first was used to check the validity and reliability, while the second was used to assess the relationship between the constructs and model fitness. The AMOS 21 version was used to estimate the CB-based SEM model.

## 5 Results

### 5.1 Demographic profile

Demographic characteristics showed that (Table 1) more than three-fourths of the participants (76.1%) were men, and the highest frequency was in the 26–30 age range. Moreover, almost 91.3% of the respondents were younger than 35 years. Most respondents had either a bachelor's or master's degree. The highest proportion of respondents (67.8%) had 1–5 years of experience. Most of the respondents were under male supervision (86.1%).

**Table 1:** Respondent's profile (N = 460).

Variables	Categories	Frequency	Percentage
Gender	Male	350	76.1
	Female	110	23.9
Age	Less than 21	1	0.2
	21–25	78	17.0
	26–30	222	48.3
	31–35	119	25.9
	36–40	25	5.4
	Above 40	15	3.3
Education	HSC	10	2.2
	Bachelor	171	37.2
	Master	270	58.7
	Above Master	9	2.0
Experience	1–5	312	67.8
	6–10	105	22.8
	11–15	27	5.9
	16–20	13	2.8
	Above 20	3	0.7
Supervisor's Gender	Male	396	86.1
	Female	64	13.9

### 5.2 Descriptive statistics

Descriptive statistics were estimated to comprehend overall TM practices and the extent of the adoption of laissez-faire leadership. Talent attraction had the highest mean score (4.30) followed by talent retention (4.01). The mean scores for talent engagement and development were 3.95 and 3.97, respectively. The mean score of laissez-faire leadership was 3.03. The correlation matrix (Table 2) indicates a significant positive correlation among the constructs, and the highest correlation exists between talent engagement and retention.

**Table 2:** Correlation matrix and descriptive statistics.

	LFL	TA	TR	TE	TD	Mean	SD
Laissez-faire leadership	1					3.03	0.830
Talent attraction	0.073	1				4.30	0.627
Talent retention	0.197^a^	0.667^a^	1			4.01	0.802
Talent engagement	0.227^a^	0.566^a^	0.720^a^	1		3.95	0.675
Talent development	0.220^a^	0.549^a^	0.660^a^	0.639^a^	1	3.97	0.789

a Significant at the 0.01 level (2-tailed).

### 5.3 The model fit measures

The association between the latent and observed variables was examined to assess the model's goodness of fit index and to depict the structural relationships. Then, a structural relationship analysis was performed to measure the linkages of the latent variables. To measure the five constructs, 28 items were used; one item from talent engagement (TE 9) was excluded because of its low factor loading. Although the factor loadings of laissez-faire leadership items were less than 0.50, every item was retained because the construct comprised of only three items. Despite the low factor loading, items under a construct can be retained if the number of items remain less than three after removal [37]. The measures of the model fit are presented in Table 3. The estimated scores of all indicators are within the cutoff value and meet the criteria for a good-fit model as recommended by Hair et al. [42] and Gaskin and Lim [40].

**Table 3:** Model fitness.

Measures	Estimates	Threshold	Interpretation
χ^2^	793.584	–	–
df	341	–	–
χ^2^/df	2.327	≤3.00	Acceptable
GFI	0.883	≥0.90	Acceptable
RMR	0.045	≤0.10	Acceptable
RMSEA	0.054	≤0.08	Acceptable
PClose	0.100	>0.05	Acceptable

Note: N = 460; GFI = Goodness-of-Fit Index; RMSEA = Root Mean Square Error of Approximation; RMR = Root Mean Square Residual; Source: Gaskin and Lim (2016).

### 5.4 The measurement model

**Fig. 2:**
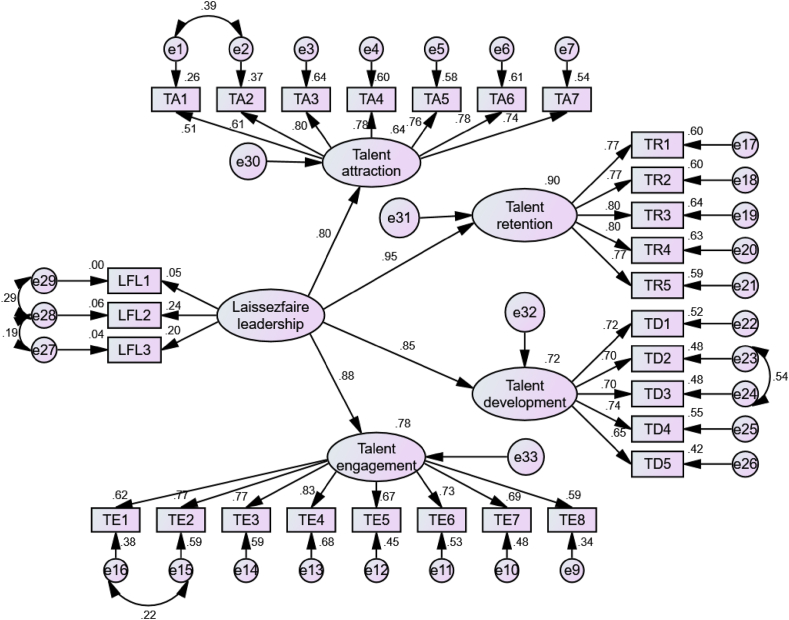
Structural model.

Factor loadings, Cronbach's alpha, AVE, and CR were used to assess the measurement model (Table 4, Fig. 2). The lowest standard loading was 0.205, whereas the highest was 0.802. The standard loading should be equal to or more than 0.50 [42]. The estimates of all four TM strategies were above 0.50, and most had a standard loading of more than 0.70. Only the items representing laissez-faire leadership had a factor loading below 0.50, but these items were not deleted, following the recommendations of Ertz et al. [37]. The minimum Cronbach's alpha value should be 0.60, but it can range from 0 to 1 [42]. The Cronbach's alpha values of the four constructs were greater than the recommended value (>0.60). The lower limit of the AVE should be 0.50, whereas 0.70 can be accepted as the CR threshold value [42]. In the present study, the AVE of the TM strategies was greater than 0.50, and that of laissez-faire leadership was near 0.50. By contrast, the CR of the TM strategies ranges from 0.827 to 0.890, and laissez-faire leadership is close to the cutoff value. Thus, the current study meets the reliability and validity criteria for all the constructs.

**Table 4:** Factor loadings, Cronbach's α, AVEs, and CRs.

Constructs	Items	Factor loadings	Cronbach's alpha	AVE	CR
Laissez-faire Leadership	LFL1	0.047	0.520	0.355	0.596
	LFL2	0.238			
	LFL3	0.205			
Talent Attraction	TA1	0.507	0.880	0.516	0.880
	TA2	0.611			
	TA3	0.802			
	TA4	0.777			
	TA5	0.764			
	TA6	0.783			
	TA7	0.735			
Talent Retention	TR1	0.773	0.887	0.612	0.887
	TR2	0.772			
	TR3	0.799			
	TR4	0.796			
	TR5	0.771			
Talent Engagement	TE1	0.618	0.890	0.506	0.890
	TE2	0.769			
	TE3	0.770			
	TE4	0.827			
	TE5	0.667			
	TE6	0.727			
	TE7	0.692			
	TE8	0.585			
Talent Development	TD1	0.720	0.827	0.489	0.827
	TD2	0.695			
	TD3	0.695			
	TD4	0.742			
	TD5	0.649

### 5.5 The structural model

This study hypothesized positive relationships between laissez-faire leadership and talent attraction, retention, engagement, and development. The standardized estimate (β = 0.803, *p* < 0.01) indicates a positive relationship between laissez-faire leadership and talent attraction; thus, the first hypothesis can be accepted. Furthermore, the remaining direct paths in this study were found to be significant. For instance, the paths from laissez-faire leadership to talent retention (β = 0.948, *p* < 0.01), talent engagement (β = 0.882, *p* < 0.01), and talent development (β = 0.849, *p* < 0.01) were all found to be significant and positive (Table 5).

**Table 5:** Result of the structural model.

Hypothesis	Stnd. Estimate	SE.	CR.	P	Decision
LFL → TA	0.803	0.455	4.813	0.000	Supported
LFL → TR	0.948	0.678	4.860	0.000	Supported
LFL → TE	0.882	0.555	4.867	0.000	Supported
LFL → TD	0.849	0.525	4.788	0.000	Supported

Note: LFL = Laissez-Faire Leadership, TA = Talent Attraction, TR= Talent Retention, TD = Talent Development, TE = Talent Engagement.

## 6 Discussion

The results reveal a different empirical perspective on laissez-faire leadership that positively influences the four major TM strategies. In other words, the study supports the idea that laissez-faire leadership facilitates the implementation of TM practices, attracts more talent explicitly, engages and develops them with a high retention rate. The first finding confirms a positive association between laissez-faire leadership and talent attraction. Although Yang [108] demonstrated a similar finding, this result contradicts those of several previous studies [13,45,52,55,88]. One possible reason for this is that employees perceive freedom and autonomy positively. Talented employees in the pharmaceutical industry want freedom, and laissez-faire leadership fulfills their expectations. Empowerment and less involvement of leaders could also explain this outcome. It is not always true that laissez-faire leadership is an avoidance-leadership approach [108]. Sometimes, subordinates expect to be left alone, so that they can work without close monitoring. They feel respected and learn to manage situations and solve problems. Consequently, the pharmaceutical industry has been attracting talented employees. Thus, laissez-faire leadership is positively related to talent attractiveness.

The second finding supports the positive relationship between laissez-faire leadership and talent retention. This result contradicts those of Ahmad et al. [2], Fedirko and Sanz [39], and Yukl [110]. Such differences can be attributed to several reasons, such as employees being accustomed to freedom in their work, thus not liking being controlled and monitored. Therefore, when they enjoy autonomy, they tend to stay there longer. Yang [108] concluded that laissez-faire leadership could enhance followers' determination and increase the likelihood of employee retention. Talented employees in the pharmaceutical industry enjoy autonomy and expect less involvement from their leaders. When employees enjoy their tasks without any intervention from their bosses, they tend to stay with the organization [95]. Under these circumstances, laissez-faire leadership is the strategic choice of the leaders, and a balanced application of this approach can yield positive results [108]. As uninvolved leaders, laissez-faire leaders create opportunities for followers to perform their tasks in ways that ultimately enhance their bonding with jobs and organizations. Consequently, the tendency to quit one's job may reduce. Moreover, under this leadership approach, subordinates are not forced through punishments or fear of retrenchment, which also influences citizenship behavior, such as a higher intention to stay. Therefore, considering these issues, the positive association between laissez-faire leadership and talent retention can be accepted.

The third finding on the relationship between laissez-faire leadership and talent engagement is positive and significant. This outcome contrasts with a few past studies [70,77,84,85], but is consistent with Thanh and Quang [101] and Yang [108]. This finding is logical for several reasons. One reason for this could be autonomy and greater control over one's job. Liu and Fu [65] stated that autonomy in the job increases talent persistence and devotion. Moreover, the opportunity for self-control removes the tension caused by the unexpected intervention of the supervisor at work. Thanh and Quang [101] argue that laissez-faire leaders' lower intervention attitudes create a culture of creativity and engagement with critical responsibility. As subordinates are less stressed owing to less involvement of the leaders, they tend to engage in more work. The high involvement of leaders may harm their engagement and satisfaction [58]. Moreover, frequent feedback or punishments lead to stress [64] that may interrupt the psychological involvement of talent. When leaders are highly supervising, there is an increased reliance on them, which ultimately reduces self-devotion toward work. Therefore, this result is justified for the pharmaceutical industry, as talent is self-determined and self-competent with less involvement from leaders [80]. Laissez-faire leaders allow subordinates to be free from controlled motivation [108], leading them to unlimited self-motivation for emotional and cognitive involvement. Hence, the outcome is justifiable in the present context, in which laissez-faire leadership is positively correlated with talent engagement.

In this study, laissez-faire leadership was hypothesized to be a positive predictor of talent development. This result is consistent with those of Eagly et al. [33], Ryan and Tipu [90], Thanh and Quang [101], and Yang [108], but inconsistent with those of Chaudhry and Javed [24], Einarsen [34], Khan et al. [56], and Skogstad et al. [96]. There are several possible reasons for this positive relationship. Laissez-faire leaders tend to be less involved in decision-making. Consequently, subordinates have more opportunities to make decisions and solve problems, which improves their decision-making skills and problem-solving capabilities. Laissez-faire leaders allow employees to solve difficult problems and assign complex responsibilities, which foster their decision-making capabilities and personal growth.

Additionally, when subordinates are left alone, self-improvement and self-directed learning are accelerated. Subordinates engage in critical self-analysis to overcome weaknesses and find creative ways when there are few instructions from the leader. Under laissez-faire leadership, subordinates are free from fear of their performance; therefore, their spontaneous involvement contributes to their self-development. As a result, new ideas can be injected into the organization, as talented employees are free to create and implement ideas. Over time, self-practice can help talented employees explore their wisdom for an organization's betterment. When subordinates are empowered, they like to think beyond their capacity, which helps improve their critical thinking. Laissez-faire leaders create an environment of independence that promotes intrapreneurship.

## 7 Theoretical and practical implications

This study has several theoretical and practical implications. From a theoretical perspective, the current study extends the limited knowledge regarding laissez-faire leadership [24,31,32,39,70,77,84] and its relationship with the four TM strategies. To date, few studies have empirically examined what the laissez-faire approach might do to facilitate TM strategies. Second, this study contributes to leadership literature by integrating SET as a theoretical lens to test the linkage between laissez-faire leadership, talent attraction, retention, engagement, and development. According to SET, employees who enjoy freedom, autonomy, delegation, and more meaningful responsibility from laissez-faire leaders will reciprocate to be more engaged, committed to learning, and serve the organization for a longer period. Third, this study revealed a positive relationship between pharma employees' perceptions of laissez-faire leadership and TM strategies. This supports laissez-faire leadership as a practical pathway for TM in an industry that mostly employs highly educated employees. Therefore, this study contributes to the extant literature by providing insight on the positive aspects of laissez-faire leadership [24,101,108] that does not empirically characterize laissez-faire leadership as zero leadership.

Regarding the practical implications, this study highlights the importance of laissez-faire leadership in implementing TM strategies. Laissez-faire can be viewed as a win-win leadership approach as it satisfies both leaders' (less involvement) and followers' (freedom in decision-making) intentions. Implementing such a leadership approach can reinforce employee motivation, self-directed learning, knowledge sharing, autonomy, self-appraisal, self-efficacy, and self-dependency, which may ultimately accelerate an organization's TM practices. Consequently, employees enjoy less supervision, monitoring, open-thinking, and creative environments. Thus, laissez-faire leadership is essential for the successful implementation of TM strategies in an organization.

From an employee perspective, subordinates perceive laissez-faire leadership as having positive potential; they develop a sense of self-worth, which contributes to a higher level of attraction and retention. Employees are persevering toward their performance, which indicates their engagement and self-development intentions. Pharmaceutical companies should understand that laissez-faire leadership can improve TM outcomes and contribute to attainment of competitive position. This study empirically proves the effectiveness of laissez-faire leadership in the Bangladeshi pharmaceutical industry. This study contributes to improving TM strategies in emerging economies by cultivating laissez-faire leadership behaviors among young talents. We recommend that management should positively perceive laissez-faire leadership and encourage them to apply this approach to skilled employees who are responsible, capable of thinking independently, and highly dedicated to accomplishing their objectives. Considering only the negative aspects may limit the acquisition of better results by using this leadership approach. Therefore, leaders should rethink laissez-faire leadership to manage talent and apply it wisely to accomplish maximum positive outcomes.

## 8 Limitations and future research directions

One limitation of this study is that the samples were collected from one metropolitan city, as pharmaceutical firms are also located in other cities of the country. Second, only one leadership style was considered, whereas more leadership approaches such as transactional, transformational, autocratic, inclusive, responsible, and democratic leadership could be used to conduct similar studies. Third, most of the respondents and their leaders were men. Hence, a separate study can be undertaken that considers supervisors’ gender as a control variable.

Previous studies have supported the negative impact of laissez-faire leadership on human attitudes; the findings of this study contradict this connotation. Hence, further studies can be performed to retest the findings in other industry contexts, such as banks, ready-made garments, tobacco, and telecommunications. Additionally, justifying the existing model from different perspectives and considering contextual variations can foster more research on TM. Future studies can also assess the causal relationship between laissez-faire leadership and behavioral outcomes, such as employee service behavior, employee commitment, organizational citizenship behavior, and employee performance, including in-role and extra-role performance. Another limitation of this study is that it measures TM using self-reporting procedures, in which biases may occur. In subsequent studies, researchers can consider simultaneous ratings by leaders and subordinates to measure TM and leadership.

## 9 Conclusion

This study examined the impact of laissez-faire leadership on talent attraction, retention, engagement, and development. The results revealed that laissez-faire leadership is neither an absent leadership approach nor always disliked by the followers. This study supports the positive effect of laissez-faire leadership on TM strategies. However, these findings contradict most prior research on the consequences of laissez-faire leadership. Study participants were mostly new generation educated workers with new mindsets and values, such as freedom and a participatory work culture. Hence, this study concludes that correctly applying this approach can produce positive outcomes and that leaders can nurture their followers more through increased freedom and autonomy. However, followers’ perceptions are essential in deciding the effectiveness of the leadership approach, and laissez-faire leadership might be considered.

## Author contribution statement

Mohammad Ali: Conceived and designed the experiments; Performed the experiments; Analyzed and interpreted the data; Contributed reagents, materials, analysis tools or data; Wrote the paper.

Muhammad Shariat Ullah: Conceived and designed the experiments; Contributed reagents, materials, analysis tools or data; Wrote the paper.

## Data availability statement

The current research has been conducted by the researchers’ own interests and funds. For this reason, data are not made easily available and accessible. The data might be available from the corresponding author uopn reasonable request.

## Declaration of competing interest

The authors declare that they have no known competing financial interests or personal relationships that could have appeared to influence the work reported in this paper.
